# Social Skills Through an Autistic Lens: A Qualitative Analysis of Young Adults’ Insights and Experiences

**DOI:** 10.1007/s10803-025-06869-x

**Published:** 2025-06-04

**Authors:** Órla Walsh, Conor Linehan, Christian Ryan

**Affiliations:** Department of Applied Psychology, University College Cork, Cork, Ireland

**Keywords:** Autism, Neurodiversity, Social skills

## Abstract

Navigating social interactions and developing effective communication strategies can pose unique hurdles for autistic people, impacting their overall well-being. This article presents a qualitative study of autistic young adults’ understanding of social skills. Social skills programs are also explored. The primary researcher conducted five semi-structured focus groups with 18 participants in total, aged 18–30 (11 men, 7 women). Our analysis adopts a qualitative approach, utilising thematic analyses. Through inductive thematic analysis we identified four key themes: (1) Social divide between neurotypes, (2) Conscious efforts in social interaction, (3) Adverse social experiences, and (4) Balancing authenticity and social adaptation in social skills programs. Participants emphasised the importance of choice and an individualised approach regarding support. While expressing a desire to develop various social skills, many participants embraced their identity and desired to improve self-advocacy. There is a need for tailored and individualised approaches in social skills programs, acknowledging the diverse experiences of autistic people. Additionally, choice and autonomy are important, aligning with the desire for self-advocacy.

**Supplementary Information:**

The online version contains supplementary material available at https://doi.org/10.1007/s10803-025-06869-x.

## Introduction

### Social Skills

Social skills are defined as the behaviours crucial for effectively navigating social environments and interactions (Gillis, 2007), which are necessary for positive interpersonal engagement (Rao et al., 2008). As defined by the DSM-5-TR (APA, 2013), an autism diagnosis is characterised by observed challenges in core social skills like social communication and interaction. These challenges include difficulties in initiating or maintaining conversations (Koegel et al., 2014) and interpreting nonverbal cues (Pelzl et al., 2023). Additionally, autism is often associated with lower executive functioning (Chen et al., 2016), affecting areas like emotion regulation, both of which directly influence social skills (Howard et al., 2023).

Social challenges for autistic children can manifest early, with traits such as reduced attention to social stimuli between 12 months and three years (Jones et al., 2016). While neurotypical (NT) children typically employ various social skills when interacting with peers, autistic children may struggle to use the same (Charlop et al., 2010). Social skills are important in their own right, but they contribute significantly to other areas of development and well-being (Humphrey & Wigelsworth, 2012). Autistic people often face more challenges in socialising than NT peers (Davis & Crompton, 2021), which can hinder the formation of meaningful relationships. Therefore, programs aimed at enhancing social skills may significantly improve the quality of life and social inclusion of autistic people (Kasari et al., 2012).

A scoping review (Black et al., 2022) found that while autistic people and their NT peers both view friendship through shared interests and regular interactions, autistic people face unique challenges, such as the pressure to conform to NT social norms. Autistic children often experience lower-quality friendships compared to their NT peers, leading to feelings of loneliness (Bauminger & Kasari, 2000). Research indicates that NT children experience increased loneliness when they have limited social interactions, whereas autistic children feel lonelier when their peers are unwilling to engage with them (Tsou et al., 2024). One study indicated that autistic primary school pupils perceived themselves as socially involved, identifying more friends than their NT peers; however, these friendships were often unreciprocated (Chamberlain et al., 2007). In contrast, many autistic children report having at least one friend and being satisfied with this (Calder et al., 2013). Some autistic pupils report having fewer opportunities to meet friends outside of school (Petrina et al., 2014), and proximity alone is insufficient for fostering social development (Wainscot et al., 2008). Nevertheless, friendship remains essential across the lifespan, rooted in the fundamental human need for social connection (Mazurek, 2013).

Autistic and NT people often struggle with communication due to differing expectations and experiences, leading to misunderstandings and negative first impressions (Crompton et al., 2020c; Sasson et al., 2017; Sheppard et al., 2015). Autistic people frequently feel better understood when interacting with fellow autistics (Crompton et al., 2020b). A scoping review on autistic peoples’ friendship experiences reveals a shared desire for companionship between autistic and NT people (Black et al., 2022).

### Current Social Skills Programs

For autistic people without intellectual disabilities, programs often target social cognition, social skills, and social motivation (Morrison et al., 2020). Social skills programs are widely used and beneficial for various groups, including those with social anxiety disorder (Beidel et al., 2014). Social skills programs are typically conducted in small groups by trained professionals or therapists and can also be implemented in school settings. Social skills programs are common for autistic people, particularly children (Dekker et al., 2014), and aim to improve peer interactions through direct, face-to-face instruction on conversation, friendship, and problem-solving skills (Soares et al., 2021).

Social skills programs can adopt a strengths-based approach, allowing autistic people to choose specific skills to focus on, thereby giving agency and aligning with their interests and abilities (Camilleri et al., 2023; Jones et al., 2021; Lee et al., 2023). Another common tool is Social Stories (Gray, 1998), which supports the development of various skills such as giving compliments and turn-taking (Almutlaq & Martella, 2018; Malmberg et al., 2015). However, challenges arise from the lack of a universally accepted definition of social skills (Rao et al., 2008), leading to inconsistencies in teaching and assessing these skills and potentially limiting the effectiveness of programs.

### Neurodiversity

The neurodiversity movement offers a new perspective on social skills by challenging conventional norms and highlighting the value of diverse interaction styles. Traditionally, social skills have been measured against normative standards that can marginalise those who do not conform, including many autistic people. The neurodiversity perspective focuses on social differences rather than deficits, aligning with the social model of disability (Hartman et al., 2023). This model recognises both the unique strengths of autistic people and the barriers they face (Sharma et al., 2012). By framing social differences as an inherent aspect of human diversity, the neurodiversity movement aims to transform societal attitudes and address obstacles that limit the potential of autistic people. This shift has led to positive changes in public perception, research, and policy (Cheng et al., 2023; Shaw et al., 2021).

### Gap in the Literature

Despite extensive research on social skills, there is a notable absence of qualitative studies asking autistic people for their perspectives, leaving their voices underrepresented. This study aims to fill that gap by gaining deeper insights into how autistic people understand and experience social skills. Understanding these lived experiences can help identify specific challenges and strengths in social interactions unique to autistic people, which is essential for developing more tailored and effective programs. Ultimately, this study seeks to create inclusive research that reflects the experiences and voices of the autistic community.

## Methods

### Participants

This study recruited 18 participants aged 18–30, including seven women and eleven men, across five separate focus groups, which was deemed as an appropriate sample size (Guest et al., 2016). The small group size was designed to minimise intimidation and allow more time for participants to articulate their thoughts (Trevisan, 2021). Data collection ceased once saturation was reached, monitored by analysing data as it was collected, and no new themes were found.

Recruitment was initially facilitated through a local service that provides support for autistic adults without intellectual disabilities. The service provides individualised support tailored to the personal and social development of autistic people who have completed secondary education. In order to increase the size and diversity of the sample, recruitment was also subsequently carried out through advertisements disseminated by a university neurodiversity society.

Five participants were diagnosed before age thirteen, nine during their teenage years, and three in their twenties. This distribution highlights that early support, often critical for effective interventions, was only available to five participants. Those diagnosed during adolescence may have faced unique challenges related to developmental changes, while those diagnosed in their twenties reflect the reality that autism can remain unrecognised until adulthood, potentially resulting in missed support during formative years.

Significantly, only five participants had experience with social skills programs, all of whom were men, with four having been diagnosed before age thirteen. This suggests a correlation between early diagnosis and access to or participation in such programs. All participants had completed secondary education, and many were attending or had attended university. Although not explicitly measured, co-occurring conditions like ADHD and anxiety were mentioned by several participants( See Table 1).

**Table 1:**
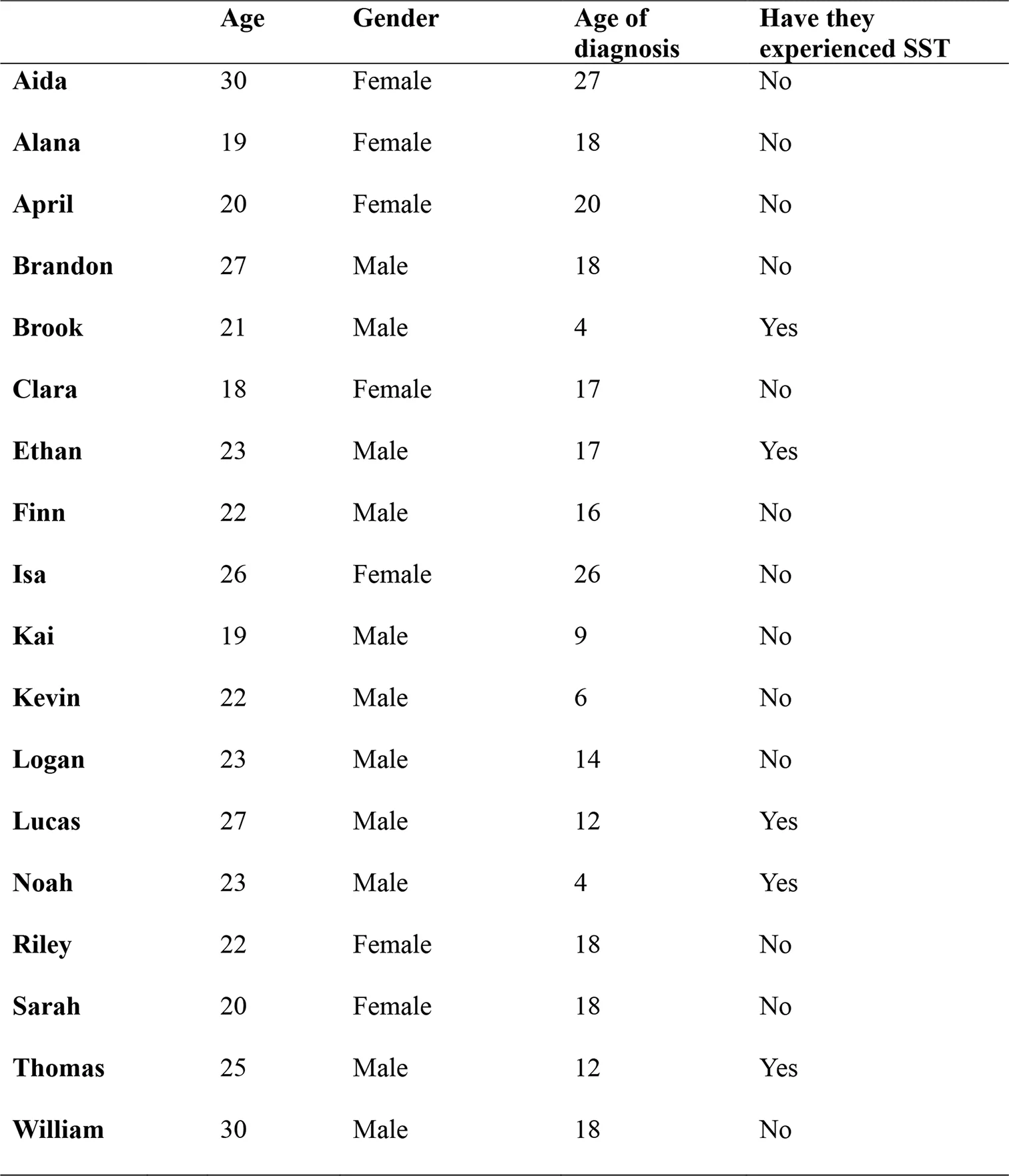
Demographic information

### Focus Groups

Participants engaged in guided discussions led by the primary researcher, with an additional author facilitating the first group. Discussions followed a series of open-ended questions. The process of writing the schedule began with a review of relevant literature. The questions were collaboratively designed by the three authors—one autistic and two NT. The guide started with simple, rapport-building topics before progressing to deeper, more specific discussions.

The group setting fostered interactive dialogue, enabling participants to challenge and expand on each other’s statements (Willig, 2008). This dynamic also encouraged participation from those who might have been hesitant in one-on-one interviews (Kitzinger, 1995). The document “Guidelines for conducting research studies with the autism community” (Gowen et al., 2019) was considered throughout.

### Method of Analysis

The qualitative method of thematic analysis (TA) was employed for open and exploratory questions. Transcripts were analysed inductively using TA to identify overarching themes. TA was selected for its effectiveness in uncovering patterns and its theoretical flexibility (Braun & Clarke, 2006; Willig, 2008). This analysis aimed to present participants’ experiences as authentically as possible, guided by a realist stance that seeks to reflect participants’ experiences as truthfully as possible.

### Data Collection and Informed Consent

Focus groups were audio-recorded. Participants were also given the option to provide written responses to questions they felt uncomfortable sharing verbally. These written responses often included additional context to supplement their verbal answers. Participants’ identities were protected using pseudonyms, and informed consent was obtained through an information sheet.

### Accessibility

A welcome pack, following guidelines by Cassidy et al. (2021) and Winn (2015), was provided. It included requests for pronouns, preferred language, and sensory preferences. Participants were encouraged to take breaks and use sensory aids, and a venue picture guide was provided to ease anxiety.

### Enhancing Reliability and Validity

To enhance reliability, data saturation was ensured (Saunders et al., 2017). Validity was strengthened by using an inductive TA, guided by the participants’ lived experiences, and by collaborating on the interview guide. The inclusion of both autistic and NT researchers reduced bias. Accessibility measures ensured participants could fully engage, allowing for richer and more accurate data.

### Reflexivity and Community Involvement

I, the first of three authors, am autistic, which provides me with a unique insider perspective. This position offers both advantages and potential biases. My personal experiences could influence my interpretation of the data, leading to an overemphasis on themes that resonate with me. To mitigate this, I kept a reflective journal throughout the research process. I also engaged in regular discussions with my co-authors to incorporate their perspectives and ensure that the analysis remained balanced and comprehensive.

## Results

Table 2 (appendix 2) presents four key themes: (1) Social divide between neurotypes, (2) Conscious efforts in social interaction, (3) Adverse social experiences, and (4) Balancing authenticity and social adaptation in social skills programs. Thirteen subthemes are also identified. These themes encapsulate the core areas explored in the study and provide insights into the experiences and perspectives of autistic people regarding social interactions.

**Table 2:** Themes and subthemes

Theme	Subthemes
Social divide between neurotypes	Unwritten rules and communication differences Emotional impact of social divide Embracing authenticity and self-advocacy Desire for autistic friendships
Conscious efforts in social interaction	Masking Mimicking Preparation rituals
Adverse social experiences	Challenges in social interaction Emotional toll of social expectations Stereotypes and their impact on relationships
Balancing authenticity and social adaptation in social skills programs	Impact of a late diagnosis Concerns with standardisation Autonomy and flexibility in program design

## Overarching Themes

### Social Divide Between Neurotypes

These narratives highlight the disparities in understanding and navigating social rules, the pressures to conform, and the resulting anxiety, self-doubt, and disconnection that is sometimes experienced. Despite this, many participants wished to embrace their differences and expressed desire to foster more autistic friendships.

#### Unwritten Rules and Communication Differences

Participants described the challenges of mismatched communication styles and the perceived imbalance in societal expectations. Participants often expressed concerns about “seeming rude” due to these differences, highlighting the underlying tension between communication preferences.

Sarah illustrated this disconnect, saying, “there’s unwritten special rules that everyone has to follow.” This comment encapsulates the struggle to navigate implicit social norms that can result in autistic people feeling disadvantaged. Sarah’s sentiment reflects a recurring theme among participants, where the invisibility of “rules” fosters feelings of exclusion and confusion. As Aria elaborated, “NTs tend to seek out hidden meanings in our words, whereas we seek directness in theirs,” underscoring the perceived bidirectional nature of this disconnect. This tension is well-documented in research, which shows that mismatched communication styles can act as significant barriers to mutual understanding (Crompton et al., 2020c).

Clara likened the social knowledge of NTs to being given a “handbook at birth,” a metaphor that captures the perceived advantage NT people may have in decoding social contexts. Ethan’s comment, “just use your common sense but…common sense isn’t always so common”, underscores the challenge he faces in navigating social norms that may seem intuitive to NTs. William added, “It’s an unfortunate reality but we don’t make the rules… they think, why can’t you be normal?”. Lucas believes that “nuances come naturally to NT people. The difficulty of navigating these norms may also permeate familial relationships. Riley expressed “it makes me really sad that they don’t like including me in things, but I think it’s literally just because I’m just not on the same… wavelength…they can just talk to each other so like, without pressure” regarding her NT sisters. This aligns with research showing that autistic people frequently feel different and disconnected from their peers (Crompton et al., 2023; Smith & Sharp, 2013). It must be noted that NT people also vary in their social skills and access to social knowledge.

The perceived imbalance in societal expectations further exacerbates these frustrations. Aria and Sarah discussed how “autistic people can be so straightforward with their intentions,” yet they feel NT people often fail to reciprocate this transparency. Aria remarked, “That is why so many NT people don’t communicate what they actually mean.” This observation may point to a broader societal bias in which autistic people are expected to adapt to NT norms without reciprocal effort. This sentiment aligns with research indicating that autistic people, particularly women, feel heightened pressure to conform to societal expectations (Yau et al., 2023).

For some participants, online communication provided a reprieve from these challenges. Noah shared that he prefers online interactions because “the normal rules of socialising don’t apply…it’s completely different because you can look over what you’ve said, what you’ve written before sending it”. This highlights the benefits of asynchronous communication, which allows for greater reflection in interactions. Logan similarly appreciated the ability to explicitly signal intent in online spaces, saying, “you can literally put in brackets -this is a joke– or -this is sarcasm- or things like that”. These remarks emphasise the role of digital platforms in reducing the ambiguity and pressure often associated with face-to-face communication.

#### Emotional Impact of Social Divide

Participants described anxiety, self-doubt, and a sense of exclusion stemming from societal pressures to conform. These feelings often contributed to a perception of being an outsider.

Thomas illustrated this sense of exclusion by comparing interactions with NT people to “window shopping” or being on the “outside looking in,” metaphorically capturing both his longing for connection and the barriers preventing it. This imagery underscores the dual experience of yearning for inclusion while feeling fundamentally separated. Riley echoed these sentiments in the context of her NT siblings, explaining, “It’s painfully obvious… I’m nothing like them,” highlighting her sense of disconnection. These reflections resonate with research on the experience of being an outsider among NT peers (Cage & Howes, 2020; Goodall, 2018).

Participants also expressed frustration at the perceived double standards in societal expectations. Logan remarked, “you’re always expected to conform to the etiquette of everyone else around you and then you start to notice how they don’t do it at all”, pointing to a potential unfairness in societal expectations, underscoring the pressure autistic people may experience to conform (Griffith et al., 2011). While these frustrations are significant, they may also resonate with NT people who feel excluded, encouraging reflection on the shared human experience of navigating social dynamics.

The emotional impact of this social divide was further evident in participants’ accounts of self-doubt. Finn shared how conversations cause his mind to “just kind of race,” leaving him opting for “short answers”. Similarly, Thomas described how “when it comes to other people, you notice there’s a lot of eyes on you and it tends to, like, make you kind of clear minded.” His description reflects a heightened self-awareness and discomfort, consistent with the cognitive overload some autistic people experience during social interactions. Noah added to the discussion, expressing feelings of inadequacy when interacting with strangers. These findings align with research indicating that up to 50% of autistic people experience social anxiety, a significantly higher rate than in the NT population (Spain et al., 2018). This heightened anxiety often leads to social withdrawal, further compounding isolation (Cappadocia et al., 2011).

#### Embracing Authenticity and Self-Advocacy

Despite these challenges, participants expressed a strong desire to embrace their identity, to advocate for their needs and move away from masking. For example, Isa wants to “advocate for myself more because I know exactly what I want and deserve”, highlighting her commitment to self-assertion and recognition of personal worth. Riley shared her goal “to not be so hard on myself anymore” emphasising self-compassion, while April added **“** I want to not see myself as less worthy than other people”. These sentiments reflect a collective aspiration among participants to reject self-doubt and societal pressures, embracing a more positive and authentic self-view.

The desire to live authentically aligns with the concept of unmasking, which is advocated by autistic pride movements and emphasises embracing one’s true identity (Weaver et al., 2021). Unmasking is not only about self-expression but also requires navigating the complexities of societal expectations, with safety often being a critical consideration (Hartman et al., 2023).

Research has shown that autistic people, both adults (Nguyen et al., 2020) and children (van der Cruijsen & Boyer, 2020), often experience lower self-esteem and self-worth. Autistic youth, in particular, are vulnerable to low self-esteem and internalising symptoms (van der Cruijsen & Boyer, 2020). However, it is important to acknowledge that many autistic people are confident and content with their identity (Cooper et al., 2020).

#### Desire for Autistic Friendships

A prominent theme among participants was to “connect more with other autistic people”. This preference stemmed from a shared understanding and ease in communication that was sometimes lacking in interactions with NT peers. While many participants described challenges in forming or maintaining NT friendships, they emphasised the unique sense of connection and mutual understanding that autistic friendships provided.

The dynamics of friendships were a key focus. Sarah described an earnest belief in people’s good intentions, stating, “you tend to put a lot of trust in people and assume that everyone has your best interests at heart”. In contrast, Riley conveyed a sense of disconnection, remarking, “you’ll never fit into that space, and they don’t even have to try”, underscoring the isolation she experiences due to perceived differences.

The appeal of autistic friendships was echoed in the shared experiences participants described. Aria shares, “I think a reason why it’s so much easier to like, converse with other neurodivergent people because you interrupt each other all the time and it’s absolutely fine”. Sarah described herself as a “neurodiversity magnet”, intuitively recognising shared traits even before her diagnosis. Similarly, Ethan states “I have a couple of autistic friends that I knew before I knew about their diagnosis… there were certain quirks of theirs that you kind of pick up on”. Logan echoed this sentiment, sharing, “There have been multiple times where… they aren’t diagnosed when they meet me and I’ll be like– oh, I’ve noticed all of these things- and then they’ll get diagnosed later”. These quotes collectively underscore the resonance and instinctive connection many autistic people find in autistic friendships.

Participants highlighted the ease of communication with other autistics. Riley observed, “it’s easier not having to explain everything”, and April expressed apprehension about NT people due to fears of being misunderstood “if you don’t get it, it’s like a really big deal”. This suggests a heightened sensitivity to misunderstandings in NT interactions. Aria captured the comfort of quiet companionship or parallel play, saying, “we’ll just sit in a room and not even speak to each other and, like, share memes”. This sentiment was echoed by April, who elaborated, “It’s much easier to just be yourself and just, like, hang out in a room without talking to each other for, like, hours”. These descriptions reveal the value placed on non-verbal connection and the mutual understanding that silence is not awkward but comforting.

These experiences align with literature highlighting the tendency to form friendships with those who share similar traits (McPherson et al., 2001; Sinclair, 2010). However, as autistic people can feel like a social minority, they may experience pressure to conform when interacting with NTs (Crompton et al., 2020a; Livingston et al., 2019).

### Conscious Efforts in Social Interaction

Participants described intentional strategies to manage and adapt to NT social norms, often through masking and mimicking behaviours, as well as preparation rituals. These strategies, while seen as necessary for fitting in, were described as cognitively demanding, emotionally exhausting, and sometimes leading to feelings of inauthenticity. The nuanced practices participants shared, reveal the complexity of navigating societal pressures to conform. The toll of these efforts was frequently described metaphorically as a “social battery,” emphasising the finite energy participants could expend in social contexts. Ethan described needing time to “recharge,” while Lucas likened socialising to the “odyssey—like going on this epic journey.” Riley believes this is because autistic people “have to think about everything ten times more than anyone else,” while Noah detailed the cumulative cognitive load, “when I’m talking to a new person, I have to build up a framework while continuously talking and receiving information, which gets tiring.”. Some participants actively avoided situations they anticipated would deplete their energy.

#### Masking

Masking was a prominent strategy participants used to camouflage autistic traits and blend into NT environments. This often involved monitoring and adjusting behaviours, suppressing natural tendencies, and adhering to expected social conventions. For instance, Isa reflected on the effort required to maintain eye contact:

I’m talking to the wall and I’m dead happy, dead confident to talk to the wall but to NT people, I make sure that I am at least like, looking at you directly for as long as I can. I never knew this was a thing with other people, that they didn’t do, that like, nobody else says, okay now look away, and now you can look at them again… nobody else does apparently but it’s all I’ve ever done.

Isa’s account underscores the cognitive load and hyper-vigilance involved in compensatory strategies. Her description illustrates how deeply ingrained these adaptations become, to the point that she only later recognised their divergence from NT norms. Similarly, Kai described the constant awareness and self-regulation masking entails,“it feels like you’re very cautious of every move you make, you know? You try to not overreach or…. being aware of everything that you’re doing, having second thoughts, and also trying not to stand out”. Logan shared, “I mask really hard all the time and so, I feel like I’m very good at a lot of the social skills that are expected of me” but acknowledged the dissonance this created, “now you’re out there and you’re not conforming to your own standards.” This reflects broader findings that masking involves adapting to a “predominantly non-autistic social world” (Cook et al., 2021, p. 1) through strategies such as suppressing stimming behaviours (Livingston & Happé, 2017). Masking is a common experience among autistic people, particularly among women (Hull et al., 2020; Lai et al., 2016; Schuck et al., 2019) and has been associated with heightened levels of stress and increased risks of suicidality (Cage & Troxell-Whitman, 2019; Cassidy et al., 2018) despite providing short-term social benefits. Efforts to adapt or mask can lead to autistic burnout, characterised by chronic exhaustion, reduced daily living skills, and an increase in autistic traits (Higgins et al., 2021; Phung et al., 2021). Although the academic exploration of autistic burnout is relatively recent, it has been discussed extensively in online autistic communities for more than a decade (Arnold et al., 2023). While most participants recounted making conscious efforts to conform to NT norms, some narratives highlight deviations from this pattern.

#### Mimicking

Many participants engaged in mimicking behaviours, deliberately copying others’ speech patterns, mannerisms, or conversational styles to fit in. Noah deliberately copies others’ speech patterns. Noah and Riley consciously copied others, with Noah being cautious when speaking and Brandon emphasising his constant second-guessing of every word. April expressed feeling like a “fake version” of herself, while Isa explains “we just go with the norm anyway and how we’ve seen everybody else act.” These narratives align with findings that autistic people often employ compensatory strategies fit in (Bargiela et al., 2016). Such strategies, while effective in achieving short-term social acceptance, are often described as double-edged swords, leading to missed opportunities for authentic self-expression (Livingston et al., 2019). Autistic women tend to compensate more frequently than autistic men (Bargiela et al., 2016; Lai et al., 2016). Assimilation, adopting behaviours or interests to fit in, is often reported as a motivation for masking among late-diagnosed autistic women (Hull et al., 2017). However, these strategies may obscure traits of autism and complicate diagnosis (McQuaid et al., 2021).

#### Preparation Rituals

Many participants developed structured routines and preparation rituals to manage social interactions. Adaptive coping mechanisms featured prominently.

Clara, for instance, articulated her efforts to learn eye contact norms, “I don’t know how long to keep eye contact with someone…, but I’ve learned to do it the best way that I can”. Diagnosed at 18, Sarah recounted developing a structured system for managing conversational pauses, “Oh my God, how wasn’t I diagnosed earlier? I developed a system that if it was quiet for three seconds, then I’d speak” While these strategies helped navigate NT social spaces, Sarah emphasised the emotional toll they exacted, describing them as stressful and exhausting. This aligns with Riley’s observation that “it’s why we’re so tired all the freaking time,” a sentiment Isa echoed by noting “and why we have so many headaches”. These reflections reveal the dual nature of adaptive strategies—enabling social navigation whilst also having consequences.

Preparation rituals further illustrate the cognitive and emotional demands of social interaction. Ethan and Brook described rehearsing conversations beforehand, while Clara created lists of questions to facilitate small talk, echoing findings from Hull et al. (2017) regarding camouflaging experiences. Logan reflected on the deliberate effort required to navigate social interactions, noting, “you have to think about your social skills and how you’re interacting with people… you’re relying on things that I don’t necessarily have the same amount of access to”, indicating that these skills might not be intuitive. This highlights the conscious processing often involved in interpreting and responding to social cues.

### Adverse Social Experiences

Participants highlighted the challenges and emotional impact of socialising, shedding light on the impact of negative perceptions and experiences. Many participants expressed feelings of isolation.

#### Challenges in Social Interaction

All participants recounted negative experiences in socialising, which often contributed to feelings of loneliness, anxiety, and social withdrawal. These experiences ranged from struggles with trust to difficulties interpreting social cues.

Many expressed a struggle with “awareness of what’s around them” reflecting the difficulties autistic people may face in interpreting verbal and non-verbal cues during interactions. Several participants expressed a general wariness towards NT people. Alana and April are cautious of NT people, staying quiet unless among close friends. This hesitancy reflects a protective strategy, which aligns with research indicating that autistic people may avoid interactions to shield themselves from potential judgment or misunderstanding (Cola et al., 2022).

Brandon shared how repeated experiences of deception during his school years led to feelings of anger, a strong aversion to school, and eventual social withdrawal. His account illustrates the impact of peer mistreatment on well-being, consistent with findings on bullying among autistic children and its association with earlier school refusal compared to NT peers (Ochi et al., 2020).

Difficulties regarding interpersonal dynamics, such as “understanding social cues and emotions” was another common challenge. Isa’s struggles hindered her ability to form meaningful relationships, while Finn described a fear of “saying the wrong thing at the wrong time” and “sometimes, I’m so unable to read the room that I say stupid things, and then people just look at me like, Who the hell is he?”. His account highlights the self-consciousness and fear of judgment that can accompany social misunderstandings. Research suggests that NTs may form less positive first impressions of autistic people, potentially influenced by misperceptions that hinder social integration (Alkhaldi et al., 2021; Sasson et al., 2017). Finn’s decision to retreat into silence after a negative experience during a game may reflect a coping mechanism to protect himself.

Skills such as “initiating a plan” and “asking people to hang out” were identified as challenging. Noah explained, “I think the hardest part and one of the most important parts is being able to initiate conversation or initiate an interaction”. Difficulties with initiation may be associated with autistic inertia, which describes the difficulty in acting on one’s intentions (Buckle et al., 2021). Autistic people can be less likely to initiate social relationships (Liebal et al., 2008), which may also be related to executive functioning (Lieb & Bohnert, 2017) or social anxiety, with approximately 40% of autistic people experiencing an anxiety disorder (van Steensel et al., 2011).

Feelings of isolation were prevalent. Ethan expressed a preoccupation with how others perceived him, while William described loneliness in school, “nobody talked to me, and I didn’t really talk to anybody.” Thomas described how leaving the classroom after class symbolised his social struggles, saying, “I was the last out—that’s kind of how it felt in the conversations”. This sense of being left behind aligns with literature on the profound loneliness experienced by autistic students (Goodall, 2018; Kasari & Bowker, 2013).

#### Emotional Toll of Social Expectations

Participants shared the emotional toll of navigating social interactions, often compounded by the pressure to conform to NT norms. These experiences frequently resulted in anxiety, burnout, and difficulties establishing healthy relationships.

Sarah characterised masking as a “survival mechanism” sharing, “I had to study what was going on around me in order to like, fit in… otherwise I was ostracised immediately… and I was being ostracised as it was”. Her account emphasises the emotional toll of constant self-monitoring and the fear of social exclusion, consistent with research linking masking to autistic burnout (Chapman et al., 2022). Lucas described overanalysing every detail, reflecting anxiety and self-criticism. This resonates with research showing autistic adults exhibit clinically significant levels of worry, surpassing those of NT adults (Black et al., 2022).

Many participants, particularly women, described experiencing certain relationships as “toxic,” conveying the negative impact on their mental and emotional well-being. Sarah explained her challenges in “negotiating boundaries and identifying red flags” which often led to harmful dynamics. Her struggles illustrate the heightened vulnerability that can arise from difficulties in social inference.

The emotional toll of navigating social spaces was particularly evident in Riley’s recollection of school, “it turns out I was like, everyone was like, laughing at me and not with me”. This memory underscores the vulnerability of those who are seen as different. These accounts align with research showing autistic women’s challenges with social inference and susceptibility to bullying (Sedgewick et al., 2019). Participants also reported experiences of friendships ending abruptly, reflecting the complexities and nuances of forming and maintaining friendships (Hickey et al., 2017). Regarding gender differences, girls regardless of diagnosis, have reported higher ‘positive friendship quality’ than boys (Head et al., 2014; Sedgewick et al., 2019).

#### Stereotypes and Their Impact on Relationships

Participants highlighted the challenges of navigating relationships and the impact of stereotypes on their self-esteem and social interactions.

Clara struggled to maintain family contact after moving to college. She mentions that her established routine—such as calling her mother every night—was disrupted when her grandparents were away for an extended period. Following their return, Clara found it challenging to resume regular communication, which led to her labelled as ‘cold and manipulative’ by her family. This highlights how deviations from established routines, often misunderstood by others, can strain relationships and perpetuate stigmatising labels. Isa echoed these sentiments, noting the damaging impact of being labelled in such ways.

Logan feared repercussions for discussing his interests, saying, “if I talk about what I care about, I’m going to be punished for that in some way…, you’re like, I need to find a way to be normal about this”.This statement underscores the internalised pressure to conform to expectations. Logan’s fear of judgment led to withdrawal, as he chose to hide his true interests to avoid rejection. Isa reinforced this experience, saying, “I learned very, very quickly that people didn’t like hearing about my interests”. These accounts emphasise the tension autistic people may feel between expressing themselves authentically and meeting societal expectations, which often results in self-censorship and social isolation.

#### Impact of a Late Diagnosis

Receiving a late diagnosis served as both a catalyst and a barrier to self-acceptance: it facilitated greater self-understanding and accommodations whilst also highlighting prior struggles with self-acceptance. Participants described both positive adaptations and the lingering challenges that stemmed from navigating social norms without earlier support or awareness.

As shared by Clara, “getting diagnosed changed my life”. Sarah now accommodates her needs, such as using noise cancelling headphones in social situations and a whiteboard to manage her days. Poignantly she shares “you were kind of… surviving before, but now you’re living like, sure, you were able to get on by but, that doesn’t mean that you were comfortable”. This highlights the difference between enduring life and actively thriving. For April, “It feels like things about you, that you didn’t understand, or other people didn’t understand… kind of…get a meaning”. Her experience illustrates how a diagnosis can serve as a lens to reinterpret past experiences, offering both explanation and closure for previously misunderstood aspects of oneself. Similarly, Aria avoids socialising in uncomfortable environments, like nightclubs, stating, “I had to get so drunk because I could not deal with the sensory stuff at all… and then it just kind of full spiralled”. Here, Aria identifies a problematic coping mechanism for socialising, which might have been mitigated with a deeper self-understanding earlier in life. Peer pressure among young people to drink must also be noted. Sarah reflects on the internalised doubts she experienced before her diagnosis,

I think I’m autistic but there would be a huge voice in my head being like, no you’re not, you’re making this up for attention and like, even though it still happens in my head, having that diagnosis has like, significantly improved that.

Her experience highlights the pervasive impact of self-doubt.

Research has found a correlation between a stronger identification with autistic social identity and higher self-esteem (Cooper et al., 2017, 2020; Corden et al., 2021). However, those with higher self-esteem may also be more likely to engage with the neurodiversity movement.

### Balancing Authenticity and Social Adaptation in Social Skills Programs

This theme highlights participants’ calls for personalised social skills programs, with concerns arising from standardised programs that fail to address individual strengths, needs, or desires. A key limitation of these programs is that they often operate under a deficit model, assuming all autistic people require the same interventions rather than recognising diverse social strengths. Central to these concerns is the tension between staying true to oneself (authenticity) while learning to navigate interactions with the neurotypical majority. Participants identified two primary issues with current approaches: (1) the redundancy of teaching skills they already possess and (2) the expectation to adopt behaviours they find irrelevant or undesirable. These concerns underscore the importance of autonomy and flexibility when designing social skills programs, ensuring that they support social development without compromising personal authenticity.

Participants such as Kai expressed the benefits they gained from social skills programs “It really helped a lot and… it helped me adapt to things that would have been overwhelming or frustrating if I didn’t get any of those trainings”. Others questioned the necessity of social skills programs altogether. Sarah, diagnosed as an adult, expressed satisfaction with her social abilities: “I’m kind of perfect the way I am… well, not perfect…. but you know what I mean? I’m good the way I am”. This sentiment indicates that while some find value in social skills programs, others may feel content with their abilities. Isa suggested incorporating diverse voices in social skills programs development to prevent assumptions, making it more affirming for each person. These findings reflect the complexity of social interactions (Zürcher et al., 2013) and the need for social skills programs to be both flexible and affirming.

#### Concerns with Standardisation

Participants noted that standardised social skills programs might cover skills they have already mastered. Brook described the approach as “generic copy and paste”, sharing, “I was able to guess what they were going to say next, and I was 100% accurate”. His criticism reflects a broader sentiment that standardised social skills programs often employs a one-size-fits-all methodology, ignoring individual contexts. Lucas emphasised recognising individual strengths and weaknesses, stating “people have difficulties and strengths in different areas and to be able to simply identify those is huge”, reflecting a fundamental flaw in standardised programs. This perspective aligns with concerns that standardised social skills programs may unnecessarily focus on already acquired skills. Logan also worried that standardisation might reduce social skill learning to mere adherence to societal norms. Brook advocated for a more targeted approach:

Stop putting it as, you know, everyone is struggling with -this- thing so we’re going to do it- this- way…. there’s no point wasting time on the bits that you don’t need help with…because that’s just going to get yourself paranoid and waste money, time, and energy that just isn’t worth it.

These concerns highlight the importance of allowing people to develop social skills in a way that aligns with their own values and needs rather than imposing uniform expectations. Participants expressed a preference for explicit, practical approaches that emphasise direct communication and actionable strategies rather than abstract, implicit social rules that may not align with their authentic selves.

#### Autonomy and Flexibility in Program Design

Another concern is that standardised social skills programs may teach skills that participants do not find relevant or necessary. Ethan suggested that this could lead to adverse psychological effects, such as “paranoia and self-doubt”. He emphasised the importance of recognising that not everyone perceives a need for change, cautioning against “putting people in boxes”, which may lead to second-guessing oneself. Ethan advocated for a modular approach to social skills programs, akin to college courses, granting autonomy to tailor learning experiences to personal needs. This modular approach aligns with a neurodiversity-affirming perspective, allowing people to select the areas they want to develop rather than assuming. This could involve an initial assessment to identify specific goals, ensuring that training remains both relevant and affirming.

Alana highlighted, “what can we do to use your skills to your advantage” rather than assuming deficits. For example, confidence-building could be supported, as higher self-esteem is linked to a stronger perception of unique abilities (Nguyen et al., 2020). Her perspective reinforces the value of tailoring social skills programs to harness individuals’ existing abilities, rather than assuming deficits. The context of social skills program implementation is also important, considering who initiated it and whether participants provided explicit consent. Ensuring that programs are voluntary and participant-led is crucial. These factors must be examined to assess how choice and autonomy impact the effectiveness of social skills programs.

## Discussion

This study explored the lived experiences and perspectives of autistic young adults regarding social skills, revealing key challenges and strategies employed in social interactions. Four key themes were discussed: (1) Social divide between neurotypes, (2) Conscious efforts in social interaction, (3) Adverse social experiences, and (4) Balancing authenticity and social adaptation in social skills programs.

The first theme, *social divide between neurotypes*, highlights a complex interplay between societal expectations and autistic experiences, revealing nuances beyond existing research. While prior studies (e.g., Crompton et al., 2020c) identify communication challenges between neurotypes, our findings show how these mismatches can drive divergent responses. The emotional toll was significant, with reports of anxiety, self-doubt, and exclusion, aligning with research showing higher rates of social anxiety among autistic people compared to NTs (Spain et al., 2018). Despite these challenges, participants emphasised a commitment to authenticity and self-advocacy, reflecting the concept of unmasking. The study also extends understanding of autistic-autistic friendships, which participants described as uniquely affirming due to shared communication styles and mutual understanding. Research supports this draw to other autistics, showing autistic people often form deeper, more supportive friendships within their community (Crompton et al., 2020a; Macmillan et al., 2018). This research reframes social success as contingent on compatibility, challenging deficit-based frameworks.

In the second theme, *conscious efforts in social interaction*, participants described their experiences of masking, mimicking, preparation rituals. Intentional strategies—such as managing eye contact and rehearsing conversations—were used to navigate NT social norms. These strategies, while aiding social integration, were emotionally taxing and cognitively demanding, often leading to burnout and feelings of inauthenticity, particularly among autistic women (Bargiela et al., 2016; Lai et al., 2016). This study adds nuance by showing that the intensity of masking varies depending on individual experiences. For example, Isa’s reflection on eye contact and Kai’s heightened self-awareness highlight the deep cognitive load of masking. Sarah’s development of structured systems to manage social pauses introduces more complex, self-constructed routines. Furthermore, participants’ descriptions of “social battery depletion” (Higgins et al., 2021) emphasises the long-term psychological consequences of sustained masking, extending research by illustrating the individualised nature and significant impact of these strategies on autistic well-being.

In the third theme, *adverse social experiences*, participants highlighted significant challenges in social interactions, often linked to past negative experiences. Social withdrawal was described as a protective strategy to avoid judgment (Cola et al., 2022). The emotional toll of these experiences was clear, as participants noted the anxiety and exhaustion associated with trying to conform to NT norms. Participants highlighted the challenges of navigating relationships and the impact of stereotypes on their self-esteem. Participants also noted struggles with setting boundaries and the lasting impact of bullying. Many noted difficulties with understanding emotions, a challenge that can be exacerbated by alexithymia, which affects an estimated 40–50% of autistic people (Berthoz & Hill, 2005). This difficulty in recognising and processing emotions may limit the ability to relate to others and further complicate social interactions (Molnar-Szakacs et al., 2020). The study also highlights challenges in maintaining relationships, particularly for women, who reported struggles with boundaries and dynamics (Sedgewick et al., 2019). A key finding was that while late diagnosis increased self-awareness and access to accommodations, it also revealed struggles with self-doubt and unmet needs. Participants with a late diagnosis found it transformative yet challenging—providing self-awareness and accommodations but also exposing self-acceptance issues that could have been addressed earlier. This underscores the need for better access to diagnosis and tailored support to address these challenges sooner.

In the fourth theme, *balancing authenticity and social adaptation in social skills programs*, participants critiqued standardised social skills programs for teaching redundant skills and enforcing strategies that they found irrelevant or undesirable. They advocated for more personalised, flexible approaches that recognise individual strengths and needs. This study highlights how standardised social skills programs can increase self-doubt and pressure to conform, affecting participants’ sense of self and autonomy. The findings stress the importance of strength-based social skills programs that respects identity, rather than focusing solely on deficits. Isa’s suggestion to include diverse voices in social skills program development aligns with the neurodiversity movement, emphasising inclusive and adaptable approaches.

The age of diagnosis appears to correlate with access to social skills programs in this sample. Specifically, five participants had experience with social skills programs, all of whom were men diagnosed before age thirteen. This suggests that earlier diagnosis may be linked to greater access to or the pursuit of social skills programs. These findings highlight that those diagnosed later in adolescence or in adulthood may face challenges in accessing timely support, which could affect their social development and experiences. These results underscore the importance of early diagnosis and support, particularly for those diagnosed in their twenties, who may miss critical interventions during formative years.

This study underscores the complex social experiences of autistic young adults, highlighting the profound impact of societal expectations, communication barriers, and a lack of tailored support. The findings emphasise the need for more personalised and inclusive approaches that prioritise authenticity and individual strengths. By fostering environments that value neurodivergent perspectives and reducing the pressure to conform to NT norms, there is potential to alleviate the psychological toll associated with masking, social anxiety, and exclusion. Ultimately, greater societal understanding and support can enable autistic people to thrive, fostering both self-acceptance and meaningful connections.

## Recommendations

Future social skills programs should prioritise inclusivity and flexibility, addressing the diverse needs and individual differences highlighted in the theme of *balancing authenticity and social adaptation in social skills programs*. The study’s findings underscore the importance of moving beyond standardised, one-size-fits-all approaches, and focusing on the autonomy and agency of participants. This means designing social skills programs that recognises each person’s strengths, communication styles, and preferences, as participants in the study specifically identified the harm of enforcing irrelevant or redundant skills.

Involving autistic people in the design and development of social skills programs is crucial, as indicated by the theme of *social divide between neurotypes* and participants’ drive for authenticity and self-advocacy. By including autistic voices in the creation of these programs, they can be more relatable and effective. This involvement could help mitigate the emotional toll and pressure to conform to NT standards, as explored in the study.

These findings have practical implications for a variety of stakeholders. For educators, incorporating autistic perspectives into social skills program development could lead to more engaging, effective, and meaningful programs, as suggested by participants’ critique of current approaches. Healthcare providers should consider the emotional and psychological impacts highlighted in the *adverse social experiences* theme, offering more comprehensive support that addresses not just social skill development, but also self-esteem, anxiety, and the emotional toll of societal expectations. Policymakers and advocates should use these insights to create inclusive policies that ensure access to timely, personalised social skills programs, especially for those diagnosed later in life, as revealed by the *impact of late diagnosis*.

## Limitations

While this study offers valuable insights into autistic young adults’ perceptions of social skills, several limitations must be considered. The recruitment of participants from specific groups may have introduced sampling bias by disproportionately including those who are more engaged with the autistic community or those who access services. This could potentially limit the generalisability of the findings. Additionally, many participants were diagnosed later in life and had limited experience with social skills programs. The shared autistic identity among participants might have shaped the topics they were willing to discuss, introducing a potential bias. Lastly, the reliance on participants’ recollections of past experiences could affect the accuracy of the data.

## Electronic supplementary material

Below is the link to the electronic supplementary material.

Supplementary Material 1
