# Supplementary material for: Social Skills Through an Autistic Lens: A Qualitative Analysis of Young Adults’ Insights and Experiences
Source: J Autism Dev Disord. 2025 Jun 4;56(11):4376–91. doi: 10.1007/s10803-025-06869-x (PMC13687222; doi:10.1007/s10803-025-06869-x)
Supplement: Supplementary file 1 [file 10803_2025_6869_MOESM1_ESM.docx]

**Appendix 1**

**Questions and activities**

Roman is a main question.

*Italics are an activity.*

Introduce yourself (name, where you grew up, and a fact about that place)

*How did you feel introducing yourself like that in front of everyone? This is written first and then participants can share as much as they wish.*

What are social skills?

What social skills are important to you? Why?

What SS do you think are important to others/neurotypicals? Why?

What social skills do you think your friends or family would like you to learn or to use more frequently?

*Here participants will be given time to write down an answer. Then they will be given the opportunity to verbally answer. (We can put these on the wall and then discuss them without the comment being attached to a person).*

Which ones would you like to learn?

What experience have you had of social skills training? (follow-up with; what was it? What was it like)

What was that experience like? What did you learn/ how did you put it into practice?

*Show the participants resources I have brought in.*

What kind of social skills training do you think is helpful?

**Appendix 2**

Thank you for considering participating in this research project. The purpose of this document is to explain to you what the work is about and what your participation would involve, to enable you to make an informed choice.

The purpose of this study is to explore autistic young adults understanding of social skills, and experiences of social skills training. Should you choose to participate, you will be asked to take part, along with 4-5 others, in a focus group, which will be facilitated by a member of the research team. This focus group will be audio recorded and is expected to take 60 minutes to complete.

Participation in this study is completely voluntary. There is no obligation to participate, and should you choose to do so, you can refuse to answer specific questions, or decide to withdraw from the focus group. Once the focus group has been concluded, you can choose to withdraw your contribution in the subsequent two weeks. However, as the transcript will be an amalgam of voices generated from the focus group audio file, it may not be possible to delete all your data.

All of the information you provide will be kept confidential and anonymous and will be available only to the researcher and the two project supervisors. The only exception is where information is disclosed which indicates that there is a serious risk to you or to others. Please be aware, however, that while we can guarantee that we will maintain confidentiality, we cannot guarantee that group members will do the same.

Once the focus group is completed, the recording will immediately be transferred to an encrypted laptop and wiped from the recording device. The data will then be transcribed by the researcher, and all identifying information will be removed. Once this is done, the audio-recording will also be deleted and only the anonymized transcript will remain. This will be stored on a cloud storage platform. The data will be stored for minimum of ten years.

The information you provide may contribute to research publications and/or conference presentations. The results of this study are to be written as a research paper.

We do not anticipate any negative outcomes from participating in this study. Should you experience distress arising from the research process, support will be provided.

This study has obtained ethical approval.

.

**Appendix 3**

I………………………………………agree to participate in this research study.

The purpose and nature of the study has been explained to me in writing.

I am participating voluntarily.

I give permission for my focus group to be audio-recorded.

I understand that I can withdraw permission to use my data within two weeks of the focus group, in which my material will be deleted from the transcript; however, for the reasons outlined above, I understand that this may not be possible. All audio files will be deleted the transcript is complete.

I understand that data from fellow participants in the focus group will be retained.

I understand that anonymity will be ensured in the write-up by disguising my identity. I also undertake to maintain the confidentiality of the focus group.

I understand that disguised extracts from the focus group (e.g. my name / location won’t be used) may be quoted in presentations and publications (e.g. article, presentation, social media publicity of the study’s findings, etc.), if I give permission below (please tick one box):

I agree to participate in this study	☐

I do not agree to participate in this study	☐

Signed:	…………………………………….	Date: ………………

PRINT NAME:	…………………………………….

**Appendix 4**

**This page of the welcome pack has the lead researchers’ picture and information.**
